# Refining a brief decision aid in stable CAD: cognitive interviews

**DOI:** 10.1186/1472-6947-14-10

**Published:** 2014-02-13

**Authors:** Karen Kelly-Blake, Stacie Clark, Katherine Dontje, Adesuwa Olomu, Rebecca C Henry, David R Rovner, Marilyn L Rothert, Margaret Holmes-Rovner

**Affiliations:** 1 College of Human Medicine, Michigan State University, East Lansing, USA; 2 College of Nursing, Michigan State University, East Lansing, USA

**Keywords:** Decision aids, Cognitive interviews, Shared decision-making, Stable CAD, Stress testing

## Abstract

**Background:**

We describe the results of cognitive interviews to refine the “Making Choices©” Decision Aid (DA) for shared decision-making (SDM) about stress testing in patients with stable coronary artery disease (CAD).

**Methods:**

We conducted a systematic development process to design a DA consistent with International Patient Decision Aid Standards (IPDAS) focused on Alpha testing criteria. Cognitive interviews were conducted with ten stable CAD patients using the “think aloud” interview technique to assess the clarity, usefulness, and design of each page of the DA.

**Results:**

Participants identified three main messages: 1) patients have multiple options based on stress tests and they should be discussed with a physician, 2) take care of yourself, 3) the stress test is the gold standard for determining the severity of your heart disease. Revisions corrected the inaccurate assumption of item number three.

**Conclusions:**

Cognitive interviews proved critical for engaging patients in the development process and highlighted the necessity of clear message development and use of design principles that make decision materials easy to read and easy to use. Cognitive interviews appear to contribute critical information from the patient perspective to the overall systematic development process for designing decision aids.

## Background

Shared Decision-Making (SDM) with patients, informed by decision aids (DAs), is increasingly recommended as a clinical practice reform to improve medical practice and patient involvement [1,2]. Decision aids are increasingly available to communicate the pros and cons of treatment alternatives. High quality DAs assure balanced presentation of the outcomes that can be achieved by competing alternatives on a population basis, and the risks and benefits of each. Decision aids in the form of patient booklets, DVDs, and websites are largely designed to prepare patients to participate knowledgeably in discussions with their providers about specific clinical decisions. Stacey et al., in a review of randomized trials, report that DAs improve patient knowledge of options, decisional conflict and decision quality, with no detrimental effects on patient anxiety or satisfaction with care or provider [3]. However, they also note that further work is needed to determine patient adherence to chosen options and to evaluate patient-physician communication while using DAs.

A growing consensus about methods to design high quality DAs suggests one key step in the process is the involvement of patients in review and development of the tools [4]. We report here the results of engaging patients in improving a prototype DA after they had used it in their own care. The results of the pilot study using the prototype have been reported elsewhere [5]. The aim of this study was to employ a cognitive interview technique to refine the DA before disseminating for use in community and academic family and internal medicine clinics.

### DA for stable CAD patients

Percutaneous coronary intervention (PCI) is commonly used in the initial management of patients with stable coronary artery disease (CAD) in the United States. PCI is a treatment that inserts a small metal or drug-eluting mesh called a stent into the partially blocked area of a heart blood vessel to keep it open. Evidence is clear that PCI improves outcomes in acute myocardial infarction (MI) patients and function and quality of life in patients whose chest pain cannot be controlled by medications. However, numerous randomized trials over the last decade and meta-analyses in patients with stable CAD have shown there is no difference in the risk of death or myocardial infarction (MI) between patients receiving PCI and medical therapy or receiving medical therapy alone [6-8]. Patients, on the other hand, even after discussion with their cardiologists, do not understand this evidence [9,10]. Whittle et al. [10], in an interview study with 1650 patients and their treating physicians following coronary angiography, report that 83% of patients expected a survival benefit and symptom improvement, even though their cardiologists did not expect a survival benefit. The authors suggest that decision aids might be helpful during decision-making about coronary catheterization for stable CAD if practice-based barriers can be resolved [11,12].

## Methods

We conducted a systematic development process to design a DA consistent with International Patient Decision Aid Standards (IPDAS) focused on Alpha testing criteria.

### Prototype development: alpha testing 1

A prototype DA was developed by literature review and consultation with nursing, cardiology, internal medicine, literacy, and communication graphics experts. The prototype was used as a patient educational intervention described elsewhere [5].

### Revised DA created

**Figure 1:**
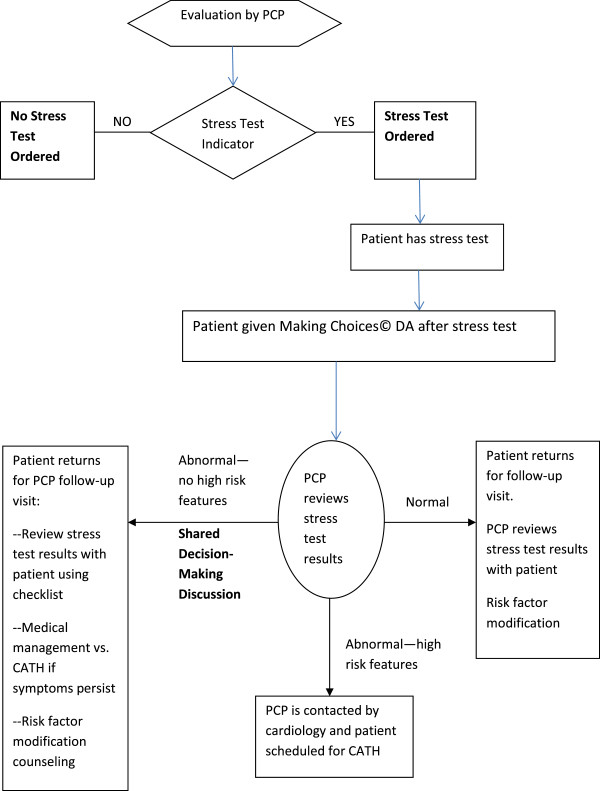
Making Choices© DA insertion point.

Based on our prior work, three important changes were made. One was to shift the introduction of the DA about decisions for and against catheterization (CATH) in stable CAD to immediately follow stress testing. A CATH is a test in which a small tube called a catheter is inserted in the arm or groin and threaded along a blood vessel until it is near the heart. When the tube gets near the heart, some dye is injected so a picture of the heart blood vessels can be seen with X-Ray. We concluded that introducing the DA at the time of CATH was probably too late for a shared decision-making discussion to occur. Our decision was to introduce the DA at a point earlier in the decision-making process which was immediately following the stress test. After the stress test, there would be questions about treatment choices and it was a good insertion point for the DA to prepare patients to participate fully in testing and treatment decisions with their providers. See Figure 1.

The second was to include the data tables about efficacy of medical therapy with angiography and stent compared with medical therapy alone in the DA. The third was to hire a graphics design firm to make the booklet more attractive. The “Making Choices©” DA provides an evidence summary and decision pages to record choices arrived at in the clinical encounter. Throughout the DA, stress test was explained as the first step in making a decision about subsequent treatment that might include whether to treat with medical therapy alone compared with medical therapy and angiography with stent in stable CAD.

### Cognitive interviewing: alpha testing 2

Cognitive interviewing is increasingly used in the development and evaluation of patient education materials [13]. Commonly referred to as “think-aloud” interviewing, cognitive interviews allow researchers to understand how patients perceive and interpret information and to identify any potential problems in the material [14,15]. The interviewee is encouraged to “think aloud” while reading through the material presented. The interviewer “probes” by asking what is confusing, or unclear, or asking for comments on specific elements in the material. The purpose of the cognitive interviews was to get feedback from stable CAD patients in order to make revisions to “Making Choices©” DA content, design, and language. The “think-aloud” approach was used by the interviewer to ask the participants what they were thinking while reading each page of the decision aid. Scripted probes were included in the interview protocol to provide content standardization across interviews [16].

### Participant recruitment

A subset of the patients from the Alpha 1 test was recruited for this study [5]. These patients, with stable CAD, had previous exposure to the DA prototype in nurse-led group visits to discuss shared decision-making with their physicians about managing their stable CAD [17]. Thirty potential participants were contacted via phone based on their previous documented interest in participating in further research. Four declined to participate, thirteen were unavailable after three attempted phone calls, and ten patients agreed to participate in the interviews.

### Development of the cognitive interview protocol

The cognitive interview protocol was developed by the research team using literature review and consensus decision-making to identify aspects of the DA for refinement. Two primary questions and seven secondary questions were developed (Table 1).

**Table 1:** Cognitive interview questions

**Question 1**	
“Please look over the first couple of pages. Take as much time as you would like to look over the pages. As you are doing this, tell me out loud any thoughts that go through your mind as you read every 1–2 pages.”	
**1a.**	**What is new or different about the information presented here from what you already know? Is there any information presented here that you question?**
**1b.**	**What have you read here that other patients might find confusing?**
**1c.**	**After reading over each section in the booklet:**
	**What is this section or part of the booklet telling you?**
	**Did it hold your attention? Why or why not?**
**Question 2**	
“What other thoughts came to mind while you were looking over the booklet that you haven’t shared?”	
**2a.**	**What do you think is missing from the booklet?**
**2b.**	**What concerns do you think other patients would have that we have not covered?**
**2c.**	**What concerns do you think other patients would have that we have covered?**
**2d.**	**What do you think the main message was?**

The interview format asked if any of the information presented was confusing, if any information was missing, what concerns patients may have, and finally what they believed was the main message of the DA. Additional probes elicited information about graphics, design, and format.

### Informed consent and IRB approval

Participants signed a consent form prior to beginning the interview. The consent form summarized the reasons for the research, protection of confidentiality and data protection procedures, and informed the participants that their participation was voluntary and that they could end the interview at any time. The institutional review board at Michigan State University approved the study protocol (IRB #09-389).

### Interviews

Interviews were conducted by a member of the research team with expertise in qualitative research (KKB). Interviews took place in a location convenient and easily accessible to the participants. The interviews were audio recorded. Each participant was given a copy of the DA. They were invited to read the pages and to “think aloud” about anything that occurred to them while reading. The questions and prompts structured these conversations. When the interviewee and interviewer had discussed all that was relevant on a page, they proceeded to the next page.

### Analysis of data

KKB audio recorded the comments of each participant and took handwritten notes of the interview. The recordings were not transcribed. KKB and SC identified common themes and concerns based on recordings and handwritten interview notes, and any differences were resolved by consensus. Our aim was to achieve thematic saturation which was reached when the participants kept repeating the same recommendations and commenting on the same concerns for each page of the DA, and as a result the data were mostly unanimous. Specific recommendations about content, design, and format were collated into a comprehensive document by SC using the audio recordings and handwritten notes. Each of the recommended changes was discussed with the research team, agreement was reached by consensus, and editing ensued. Recommendations that were not enacted were primarily due to cost and time constraints. The data from the cognitive interviews were used to refine a new version of the DA [18].

## Results

### Participants

All ten patients who agreed to participate were interviewed. They included one female and nine males. Interviews took about an hour to complete. Participants ranged in age from 60–78 years (mean 68.4; SD 6.87), were college-educated, and self-identified as White. They were all patients at the academic medical practice. The main themes that emerged from the cognitive interviews are shown in Table 2.

**Table 2:** Themes

**Identified theme**	**Example comment**
Summary feedback	Booklet was easy to understand
	Medical terms could be confusing
Content	More elaboration on prevention
	Talking Points (Figure 2) form helpful and convenient
Language	Easy read, but glossary was helpful
	Acronyms (PCI, CATH) and use of “provider” was confusing
Appearance	Difficulty reading text on colored pages
	People looked too happy
Missing information	How to determine which provider to see for management of heart disease (Figure 3)
	Clarification that booklet is a *supplement* to and not a *replacement* for physician recommendation
Main message	Multiple options to be discussed with physician
	Take care of yourself
	Stress test is the gold standard**

**This was definitely not the message we sought to convey in the DA because it was not consistent with the evidence. Revisions were made to clarify this point.

### Summary feedback

Overall, the DA was found to be logically organized and presented in a way that would allow patients to have a meaningful and appropriate conversation with their physician about the pros and cons of different treatment options. Participants found the booklet easy to understand based on their personal experience with stress tests and heart attacks. They noted that some of the medical terms could be confusing for newly diagnosed heart disease patients (a glossary page of medical terms was in the DA), but overall, the booklet would be helpful.

### Content

**Figure 2:**
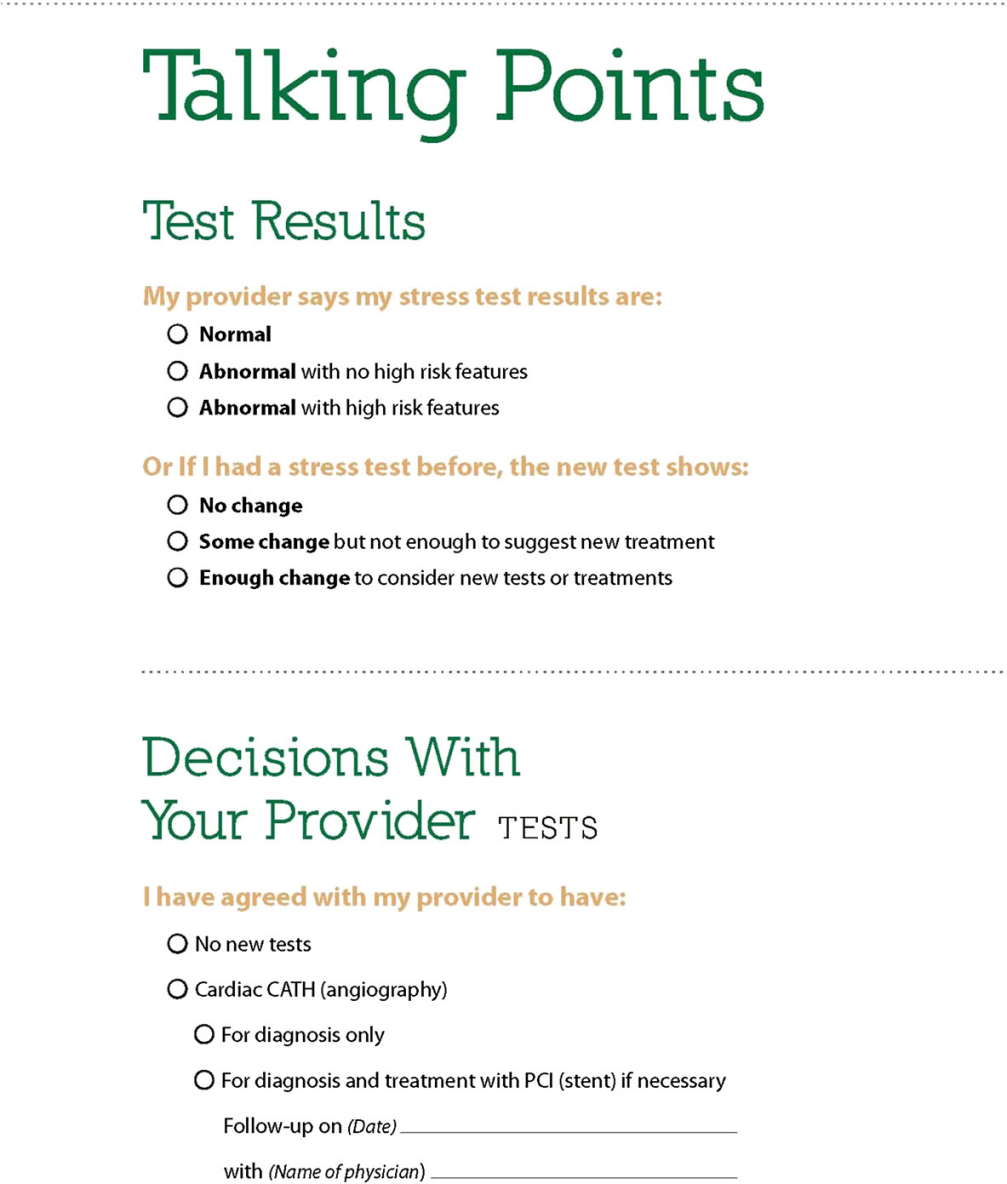
Talking Points.

Participants wanted more elaboration on the prevention of heart disease, for example, healthful diet, exercise, and smoking cessation. A few participants wanted graphs that showed data extending beyond the five year survival interval because they were concerned that the meaning was that five year survival was the best they could expect. Participants found the tear-off “Talking Points” form helpful and convenient for keeping track of issues that they wanted to discuss with their physician. Moreover, they thought the form would help commit the patient to thinking about the treatment choices (Figure 2).

### Language

Largely, participants felt the DA was an easy read. However, they tended to return to the glossary page multiple times, which they recognized as a helpful resource in the booklet. Acronyms (i.e. PCI, CATH) were confusing and use of the term “provider” was problematic because they felt it could be a reference to an insurance provider. This was easily corrected by using the term “healthcare provider.” It was stressed that because the DA would likely be read during an emotionally charged time, i.e. receiving stress test results, confusing language should be avoided.

### Appearance

Participants did not take issue with the graphics or design, but had difficulty reading the text on colored pages. They also had difficulty distinguishing the information presented in the graphs because of the colored background. Changes were made to the colors used in the booklet based on this feedback. Several participants commented that the pictures used in the booklet showed people who looked happier than most patients would be in the given situation.

### Missing information

**Figure 3:**
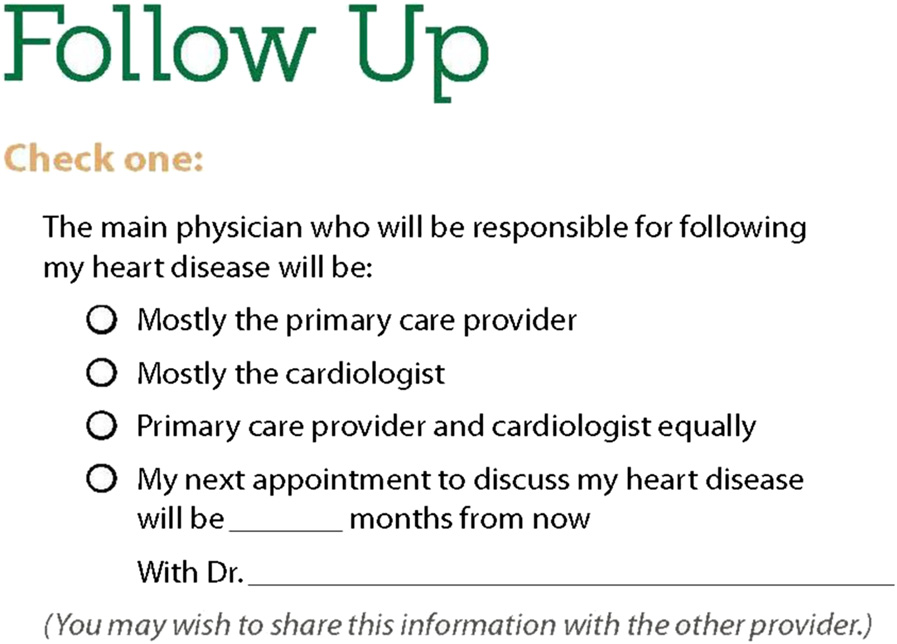
Follow-up form.

The interviewer specifically asked: “What do you think is missing from the booklet?” Participants identified several areas that needed expansion or needed to be added:

1) How to determine which provider to see regarding their heart disease (in the follow-up form, see Figure 3, they have a checkbox for their PCP, cardiologist, or both).

2) Clear statements that the booklet is not a *replacement* for speaking with a physician, but *supplements* a physician’s recommendation.

3) Information on what a spouse or family member can expect when a loved one is making treatment decisions about heart disease.

4) Clarification about the different kind of stress tests a heart disease patient may have.

### Main message

Three main messages came to light for participants: 1) Patients have multiple options and strategic choices to make based on the results of a stress test, and these choices should be discussed with a physician; 2) take care of yourself; and 3) a stress test is the gold standard for determining the severity of your disease. Item 3 was definitely not the message we sought to convey in the DA because it was not consistent with the evidence. Revisions made based on the results included the following:

• Confirmed main message, but revised to correct inaccurate assumption about the stress test as gold standard.

• Added a checklist for recording decisions such as scheduling appointments, test results, and future tests presented in a format that could go to the patient, to the clinician, and to the electronic health record. The rationale for this revision, in part, was that even a brief DA booklet is too long for the clinical encounter.

• In terms of the feedback received about missing information (see last section), text was added for all but one of the suggestions. No action was taken on suggestion four because the focus was on the exercise treadmill stress test only, and we wanted to avoid confusing the different types such as nuclear, ECHO, and chemical stress testing.

All revisions were reviewed and agreed upon by the research team. The local graphics design firm finalized the design of the “Making Choices©” DA. The DA was copyrighted and printed for distribution.

### Further development

Our Beta 1 and 2 testing (field testing) is currently underway. We have distributed the brief DA to four primary care practices and one cardiology practice in Michigan. Through this development process, we have focused on a pure primary care strategy. However, next steps in implementation will facilitate collaboration with cardiology in order to improve referral and handoffs between primary care and cardiology.

## Discussion

The study reported here used cognitive interviewing to help guide the development and refinement of the “Making Choices©” decision aid. The DA operates as an evidence summary and decision pages to record choices arrived at in the clinical encounter. The data from the interviews afforded the researchers the opportunity to include new content or modify and expand existing content. Cognitive interviews are increasingly used in the development and evaluation of patient education materials and different protocols are used [13,19-21]. Commonly referred to as “think-aloud” interviewing, cognitive interviews allow researchers to understand how information is perceived and interpreted and to identify any potential problems in the material [14,15]. We used this technique to refine the information contained in the “Making Choices©” DA before disseminating to patients and providers for use in routine clinical visits.

Our focus, reported here, was on the Alpha testing element of the IPDAS development process, which is still a work in progress. Cognitive interviews offer an opportunity as a first stage assessment within the Alpha testing process to meet IPDAS criteria. Coulter et al. [4] report that only about 50% of patient decision aids have been field tested with patients and even fewer have been reviewed or tested by clinicians. The prototype was developed in consultation with various medical professionals. The revised DA was then subjected to critique and feedback with patients previously exposed to the prototype. This iterative Alpha testing process, to have patients critique and provide feedback on the comprehensibility and acceptability of the DA, assures the validity and reliability of the development process and the final product. The cognitive interview approach lends itself well to achieving these goals. As we move forward with Beta testing, it will be important to evaluate how the approach might be useful in the field environment.

### Limitations

The cognitive interviews provided valuable input for the revision of the DA, but there are limitations to the approach. First, the interviewees were not a representative sample of all possible stable CAD patients. We interviewed a subset of patients from the larger study that agreed to be contacted for future research. These participants had previously shown a willingness to participate in research and so may be more inclined to participate in such activity. Although we cannot eliminate this limitation, one way to mitigate it is to recruit participants from diverse economic, educational, and cultural backgrounds. Additionally, our sample of participants were all college-educated and above so participants with low literacy and numeracy would be invaluable in helping assure the accessibility and understanding of the information contained in the DA. A further limitation is that the participants in this study had been exposed to a previous version of the DA to facilitate decision making with their physicians, but they were not, at the time of this study facing the decision again. Second, it was relatively easy to make some changes to the DA, especially those that focused on design, appearance, and even some of the content. Some of the feedback highlighted problem areas that were not so easy to address such as including graphs depicting data that extended beyond a five-year survival rate. The graphs included in the DA were part of a package of materials and the data included in the DA were the data available to the research team at a particular point in time. Third, the participants may have not been as critical as they would have been with an unknown interviewer. The participants knew the interviewer (KKB) from the larger study. They may have felt some affinity and did not want to seem overly critical or judgmental of the DA. Along that same vein, people agreeing to provide feedback on the revised DA may have done so because they had more favourable experiences in the original study and were therefore more likely to provide positive feedback towards this DA. Lastly, the results provided too little to fill the knowledge gaps about patient adherence to decision choice or patient-physician communication while using DAs.

In contrast, the strength of the approach was that the same themes and comments appeared in each interview, and by the tenth interview we achieved thematic saturation and there were no new issues raised.

Further evaluation is needed to determine the usefulness of cognitive interviewing as an effective approach for assuring content and message relevance in decision aids to be used by both patients and providers in routine clinical visits.

## Conclusions

The cognitive interview approach was instrumental in refining the “Making Choices©” DA. The technique proved to be critical for engaging patients as experts because their perspective cannot be provided by DA developers or clinicians. To our knowledge, this is the first study to report that stable CAD patients have been involved in cognitive interviews to refine a decision aid that addresses stress testing. Participants provided useful feedback on the design, language, and message construction. The feedback received facilitated the dissemination of the new version into community and academic practice settings to be used by both patients and providers in real world clinic settings.

Stable CAD and its concomitant treatment trajectory is a challenging clinical problem for shared decision-making and DA developers invested in producing balanced information. Cognitive interviews appear to contribute critical information from the patient perspective to the overall systematic development process for designing decision aids.

## Competing interests

The author(s) declare that they have no competing interests.

## Authors’ contributions

KKB conceptualized the project, collected the data and led the analysis, and drafted the manuscript. SC analysed the data, contributed to literature searches, and drafted parts of the manuscript. MHR contributed to conceptualization of the initial project and contributed to literature review. KD, AO, RCH, DRR, MLR and MHR critically reviewed and revised many versions of the drafted manuscript. All authors read and approved the final manuscript.

## Pre-publication history

The pre-publication history for this paper can be accessed here:

http://www.biomedcentral.com/1472-6947/14/10/prepub
